# Pattern synthesis of linear and ring arrays with minimum number of elements using FFT and Bessel transformation

**DOI:** 10.1038/s41598-022-09560-8

**Published:** 2022-03-31

**Authors:** Mahdi Boozari, Mohammad Khalaj-Amirhosseini

**Affiliations:** 1 Electrical Department, Ferdwosi University of Mashhad, Mashhad, Iran; 2 School of Electrical Engineering, Iran University of Science and Technology, Tehran, Iran

**Keywords:** Engineering, Electrical and electronic engineering

## Abstract

In antenna engineering, to reduce the final cost of array design, it is often necessary to design an array with the minimum number of elements. So, proper selection of the number of array elements and the location of the elements are two important factors in array design. In this work, a novel analytical approach for designing an array with optimal parameters is described. The Nyquist–Shannon sampling theorem is used to determine the number of array elements and the distance between them. The array's excitation coefficients are then determined using the recursive least square approach and the Bessel transform of the array factor. It is also demonstrated that the introduced procedure can be extended to include the concentric ring arrays. Several practical arrays are evaluated to verify the performance of the suggested approach. The results show that the introduced approach is a good candidate for designing practical arrays with acceptable accuracy.

## Introduction

An antenna array consists of a set of antennas. All antennas will be of the same type and size. Antenna arrays are preferable to single antennas for directive characteristics. Electronically steerable antenna arrays have an extensive range of applications, from radar to 5G communication^1–3^. Antenna arrays have advantages such as fast scan speed in all directions of space, high gain, and no mechanical components. Phased arrays use the amplitude and relative phase difference between the signals fed into properly spaced antennas to obtain the desired radiation pattern^4,5^.

The geometry of a phased array, the distance between two adjacent elements, the magnitude and phase of the array elements' weights, and the relative pattern of individual antenna elements are the few controls used to manage an antenna array's radiation pattern. For a prescribed radiation pattern, synthesis methods are used to calculate the unknown parameters of the under-studying arrays, such as excitation coefficients. Various analytical, iterative, and algorithm-based methods are introduced to synthesize the prescribed radiation pattern of equally or unequally spaced arrays. These methods can not only be used to design an array with an arbitrary radiation pattern^6,7^ but can also be used to reduce the low side lobe levels^8,9^.

The most well-known analytical methods include Fourier Transform (FT) and Woodward-Lawson technique^10^, Pattern Integrating (PI) approach^11,12^, Deterministic Space Tapering (DST) Technique^13^, Eigen-Vector Decomposition (EVD)^6^, Fourier’s Coefficients Equating Method and Bessel transform^14^, K-Means solution^15^ and Least Square Estimation Error^7^. Also, various types of evolutionary algorithm-based methods such as Genetic Algorithm (GA), Particle Swarm Optimization (PSO), and Differential Evolution Algorithm (DEA) are introduced in the literature for this target^16,17^. The pros and cons of the currently available techniques in the literature show that the synthesis of array patterns is still a challengeable problem. The main challenge in using these methods is that the number of array elements as well as the element spacing must be known before starting the synthesis process. In other words, these methods cannot be used to determine the array elements and element spacing. Moreover, some of the analytical methods cannot control the unshaped region of the desired pattern. Additionally, the algorithm-based methods are time-consuming and don’t present a clear relationship between the input and output parameters of the problem.

Due to the mutual coupling effect and high directivity, a well-designed array has the fewest number of elements and the greatest distance between them. This paper presents an analytical method to design a linear and concentric ring array with optimum parameters. It is shown that using the principle of the Nyquist–Shannon sampling theorem, the minimum value of the array elements can be determined. In addition, the upper-band of the distance between two elements is determined by taking into account the grating lobe effect. Following the specification of these parameters, the array's excitation coefficients are determined using the Bessel transform of the array factor and the recursive least square approach. It is demonstrated that not only can the proposed method be used to develop linear arrays, but it can also be utilized to design concentric ring arrays. Several practical arrays with various radiation patterns are examined to verify the performance of the suggested technique. The results show that the introduced method is a good candidate for designing the practical arrays with acceptable accuracy.

## Theory and formulation

The array factor of an equally-spaced linear array with *N* number of elements is given by Eq. (1), in which parameters *d*, *k*, *θ*, and *λ* are the distance between two adjacent elements, propagation constant defined by 2π/*λ*, elevation angle and wavelength respectively and *u* = cos(*θ*)^18^.

1 $$ F\left( u \right) = \sum\limits_{n = 1}^{N} {I_{n} \exp \left( {jnkdu} \right)} $$

The excitation coefficient of the nth element is displayed by *I*_*n*_. The target of the synthesizing problem of an equally-spaced array is to find the complex excitation coefficients *I*_*n*_’s for *n* = 1, 2,…, *N*. According to the superposition principle, the radiation pattern of an array is proportional to the summation of the radiation pattern of several isotropic elements. In^19^, it is shown that the relation between the array factor and elevation angle *θ* can be expressed in ultra-spherical polynomials. The ultra-spherical polynomial chosen for this purpose should be capable of synthesizing both sum and difference patterns. The Bessel function is considered to be a good candidate for this target. In addition, the Bessel transform can be used as an integral transformation similar to the Fourier transform, which is a special case of the Jacobi transform^20^. To this end, the first kind of Bessel function of order *p* is considered as the kernel of the transformation. For an arbitrary function *f*(*x*) over the interval *x*_*min*_ ≤ *x* ≤ *x*_*max*_, the Bessel transform is expressed as follows.

2 $$ L_{m} \left\{ {f\left( x \right)} \right\} = \int_{{x_{\min } }}^{{x_{\max } }} {J_{p} \left( {mx} \right)f\left( x \right){\text{dx}}} $$

where *J*_*p*_, *L*_*m*_ are the first kind of Bessel function of order *p* and Bessel transform of order *m*, respectively.

According to Eq. (2), the Bessel transform of the array factor is determined using multiplying both sides of Eq. (1) by the first kind Bessel function of order *p*.

3 $$ J_{p} \left( {mu} \right)F\left( u \right) = \sum\limits_{n = 1}^{N} {I_{n} J_{p} \left( {mu} \right)\exp \left( {jnkdu} \right)} $$

Since 0 ≤ *θ* ≤ π, the variable *u* becomes tightly bound over the interval −1 ≤ *u* ≤  + 1. Hence, the Bessel transform of the array factor *F*(*u*) is obtained as.

4 $$ \int_{ - 1}^{ + 1} {J_{p} \left( {mu} \right)F\left( u \right){\text{du}}} = \sum\limits_{n = 1}^{N} {I_{n} \int_{ - 1}^{ + 1} {J_{p} \left( {mu} \right)\exp \left( {jnkdu} \right){\text{du}}} } $$

According to Eq. (4), *M* equations can be extracted by varying *m* = 1, 2, …, *M*. So, Eq. (4) can be written in matrix form given by (5).

5 $$ {\mathbf{AX}} = {\mathbf{B}} $$

where,

6 $$ {\mathbf{A}} = \left[ {A_{mn} } \right]_{M \times N} \to A_{mn} = \int_{ - 1}^{ + 1} {J_{p} \left( {mu} \right)\exp \left( {jnkdu} \right){\text{du}}} $$

7 $$ {\mathbf{B}} = \left[ {B_{m} } \right]_{M \times 1} \to B_{m} = \int_{ - 1}^{ + 1} {J_{p} \left( {mu} \right)F\left( u \right){\text{du}}} $$

8 $$ {\mathbf{X}} = \left[ {\begin{array}{*{20}c} {I_{1} } & {I_{2} } & \cdots & {I_{N} } \\ \end{array} } \right]^{T} $$

By assuming that **A** is a left-invertible matrix, and also *M* ≥ *N*, the exciting vector **X** can be determined using Least Square Method (LSM) as follows^11^.

9 $$ {\mathbf{X}} = \left( {{\mathbf{A}}^{T} {\mathbf{A}}} \right)^{ - 1} {\mathbf{A}}^{T} {\mathbf{B}} $$

The calculated *I*_*n*_’s using (9) may not be accurate enough for some cases^7^. It is shown in^7^ that the conventional LSM does not have sufficient accuracy in the problem of pattern synthesis. Hence, to increase the accuracy of the final solution, the Recursive Least Square Estimation (RLSE) method can be used instead of LSM^21^. To this end, it is assumed that *M*_*0*_ number of samples of the prescribed pattern is available. *M* samples should be considered to improve the accuracy of the final solution in such a way that *M* > *M*_*0*_. As a result, RLSE equations can be written as^21^.

10 $$ {\mathbf{A}}_{m + 1} = \left[ {\begin{array}{*{20}c} {{\mathbf{A}}_{m} } \\ {{\mathbf{a}}_{m + 1}^{T} } \\ \end{array} } \right], \, m = 1, \, 2, \, ..., \, \left( {M - M_{0} } \right) $$

11 $$ {\mathbf{a}}_{m + 1} = \left[ {\begin{array}{*{20}c} {e^{{jkdu_{{m + M_{0} }} }} } & {e^{{jkdu_{{m + M_{0} }} }} } & \cdots & {e^{{jkdu_{{m + M_{0} }} }} } \\ \end{array} } \right]^{T} $$

12 $$ {\mathbf{B}}_{m + 1} = \left[ {\begin{array}{*{20}c} {\mathbf{B}} & {{\mathbf{B}}_{m} } \\ \end{array} } \right]^{T} , \, {\mathbf{B}}_{m} = F\left( {u_{{m + M_{0} }} } \right) $$

According to (6), (10), **A**_m_ = **A**. So, the excitation coefficients of the array can be calculated in a recursive form as.

13 $$ {\mathbf{I}}_{m + 1} = {\mathbf{I}}_{m} + {\mathbf{P}}_{m + 1} {\mathbf{a}}_{m + 1} \left\{ {{\mathbf{B}}_{m + 1} - {\mathbf{a}}_{m + 1}^{T} {\mathbf{I}}_{m} } \right\} $$

where,

14 $$ {\mathbf{P}}_{m + 1} = {\mathbf{P}}_{m} - {\mathbf{K}}_{m + 1} {\mathbf{a}}_{m + 1}^{T} {\mathbf{P}}_{m} $$

15 $$ {\mathbf{P}}_{m} = \left( {{\mathbf{A}}_{m}^{T} {\mathbf{A}}_{m}^{ - 1} } \right)^{T} $$

16 $$ {\mathbf{K}}_{m + 1} = {\mathbf{P}}_{m} {\mathbf{a}}_{m + 1} \left( {{\mathbf{a}}_{m + 1}^{T} {\mathbf{P}}_{m} {\mathbf{a}}_{m + 1} + {\mathbf{1}}} \right)^{ - 1} $$

It should be noted that the integral result of (6) and (7) cannot be expressed in closed-form, and numerical techniques should be used to compute them. However, using today’s computers, the required time is about a few milliseconds.

**Figure 1:**
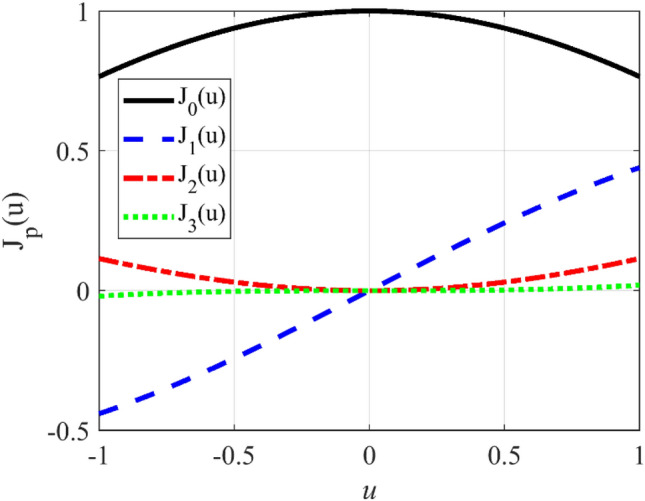
The Bessel functions of the first kind.

Figure 1 shows the Bessel functions of the first kind of orders *p* = 0, 1, 2, 3. It is seen that only for *p* = 0, the value of the Bessel function is non-zero at *u* = 0, and for *p* ≠ 0, the value of the Bessel functions at *u* = 0 is zero^22^. For this reason, and in synthesizing procedure, it is better to use *J*_0_(*mu*) and *J*_*p*_(*mu*); *p* ≠ 0 for the cases that the desired array factor has not a null in broadside direction (sum pattern), and the desired array factor has a null at *u* = 0 (difference pattern), respectively. In other words, Eq. (7) should be modified as.

17 $$ B_{m} = \int_{ - 1}^{ + 1} {J_{p} \left( {mu} \right)F\left( u \right){\text{du}}} \, \to \, \left\{ {\begin{array}{*{20}c} {p = 0} & {\text{Sum Pattern}} \\ {p \ne 0} & {\text{Difference Pattern}} \\ \end{array} } \right. $$

The presented procedure is general, and it suffices only to hold the Dirichlet condition for Eq. (7) as follows.

18 $$ \left| {\int\limits_{ - 1}^{ + 1} {F\left( u \right)J_{p} \left( {mu} \right)du} } \right| < \infty $$

It is stated in^23^ that the Dirichlet condition is fulfilled for most practical arrays.

## Determining the minimum number of array elements

In array antennas, designing an array with the minimum number of array elements is an important challenge. In this section, a new method based on the Nyquist–Shannon sampling theorem is introduced that can be used to design an array with a minimum number of elements. It is assumed that the array factor of Eq. (1) has a Fourier transform *F*_*f*_(j*ω*)^24^.

19 $$ F_{f} \left( {j\omega } \right) = \int_{ - \infty }^{ + \infty } {F\left( u \right)\exp \left( { - {\text{j}}\omega u} \right){\text{du}}} $$

Since −1 ≤ *u* ≤  + 1, then, *F*(*u*) can be considered as a band-limited signal with *F*_*f*_(j*ω*)≈0 for |*ω*|≥ *ω*_*M*_^25^. According to the sampling theory, array factor *F*(*u*) can be uniquely reconstructed by its samples *F*(*qT*), *q* = 0, ± 1, ± 2, …, if^25^.

20 $$ \omega_{S} = \frac{{2{\uppi }}}{T} \ge 2\omega_{M} $$

where, *T*, *ω*_*S*_ are sampling period and sampling frequency, respectively. We know that the closed-form formula of *F*_*f*_(j*ω*) is as follows^24^.

21 $$ F_{f} \left( {{\text{j}}\omega } \right) = \sum\limits_{n = 1}^{N} {I_{n} \sqrt {2{\uppi }} {\updelta }\left( {\omega - knd} \right)} $$

where δ(.) is the Dirac delta function. It is obvious from Eq. (21) that *ω*_*M*_ = *kNd*. Also, by remembering the sampling period *T*, the array factor *F*(*u*) can be reconstructed using the *M* + 1 number of samples over the interval −1 ≤ *u* ≤  + 1. So, we have.

22 $$ M = \frac{{u_{\max } - u_{\min } }}{T} + 1 = \frac{2}{T} + 1 = \frac{{2\omega_{M} }}{\pi } + 1 $$

By comparing Eqs. (20), (22) and *ω*_*M*_ = *kNd*, we have.

23 $$ M = \frac{4Nd}{\lambda } + 1 $$

It is seen from Eq. (1) that the total length of array *L* is determined as follows.

24 $$ L = \left( {N - 1} \right)\frac{d}{\lambda } $$

The array length *L* can be specified by the designer. As a result, the following equations can be used to determine the minimum values of *N* and *d* for a desired radiation pattern *F*(*u*).

25 $$ \frac{d}{\lambda } \simeq \frac{M - 1}{4} - \frac{L}{\lambda } $$

26 $$ N \simeq \frac{M - 1}{{M - 1 - 4\left( {L/\lambda } \right)}} $$

The obtained equations provided the optimum values of *N* and *d* for an equally-spaced array with a specified length *L* and the desired pattern *F*(*u*). However, it is stated in^18^ that for an array with its peak at *θ*_*0*_, the maximum value of *d* is obtained from the following equation so that the grating lobe does not appear.

27 $$ d_{\max } \le \frac{\lambda }{{1 + \left| {\cos \theta_{0} } \right|}} $$

If the obtained value for *d* from Eq. (25) does not satisfy the condition of Eq. (27), the designer can achieve the desired goal by changing the values of *L* or *M*. Additionally, the minimum value of array length can be achieved by substituting Eq. (27) into (25).

28 $$ \frac{L}{\lambda } \ge \frac{M - 1}{4} - \frac{1}{{1 + \left| {\cos \theta_{0} } \right|}} $$

Regarding Eqs. (26) and (28), the minimum value of array elements is determined as follows.

29 $$ N \ge \frac{{\left( {M - 1} \right)\left( {1 + \left| {\cos \theta_{0} } \right|} \right)}}{4} $$

Using (20) and (22), it can be concluded that the minimum value of array elements is limited by the frequency bandwidth of the Fourier transform of the prescribed array factor and *θ*_*0*_ as follows.

30 $$ N \ge \frac{{\omega_{M} }}{2\pi }\left( {1 + \left| {\cos \theta_{0} } \right|} \right) $$

It should be noted that Eq. (28) is used to determine the minimum values of array length, and Eq. (29) is used to calculate the minimum number of array elements corresponding to Eq. (28). Equations (28), (29) can be used for the cases where the array length is unknown, but Eq. (30) is used for the cases where the array length is predetermined. Moreover, Eq. (28) can be useful for the arrays with the continuous line-source distribution.

As a result, the proposed method can be summarized as follows.

1. According to Eq. (19), the Fourier transform *F*_*f*_(j*ω*) of the prescribed radiation pattern is calculated.
2. From the calculated Fourier transform, the suitable value of *ω*_*M*_ can be determined.
3. The value of *N* is calculated using Eq. (30).
4. The value of *d* is determined using Eqs. (24), (27).
5. The excitation coefficients *I*_*n*_’s are calculated using Eqs. (5) to (9).

## Extension to the concentric ring arrays

The presented procedure can also be used for concentric ring arrays. The array factor of a concentric ring array is expressed as follows^26^.

31 $$ F\left( u \right) = \sum\limits_{n = 1}^{N} {I_{n} N_{n} J_{0} \left( {kR_{n} u} \right)} $$

where *R*_*n*_, *I*_*n*_, *N*_*n*_, *N* are the radius of *n*th ring, elements weights of ring *n*, number of elements in ring *n* and the number of rings, respectively, and *u* = sin(*θ*). The radiation pattern of a concentric ring array is independent of azimuth angle *φ*, and all elements in the same ring have the same weight^24^. A concentric ring array can be created using the same procedure as linear arrays. To this end, it is sufficient to rewrite Eq. (6) as follows.

32 $$ {\mathbf{A}} = \left[ {A_{mn} } \right]_{M \times N} \, \to \, A_{mn} = \int\limits_{ - 1}^{ + 1} {J_{p} \left( {mu} \right)N_{n} J_{0} \left( {kR_{n} u} \right)du} $$

It is worth noting that the orthogonality of Bessel functions is given by.

33 $$ \int_{0}^{{x_{0} }} {xJ_{p} \left( {mx} \right)J_{p} \left( {nx} \right)dx} = \left\{ {\begin{array}{*{20}c} 0 & {n \ne m} \\ {\frac{{x_{0}^{2} }}{2}\left[ {J_{p} \left( {nx_{0} } \right)} \right]^{2} } & {n = m} \\ \end{array} } \right. $$

By comparing (32) and (33), it can be concluded that the orthogonality of the Bessel function has no effect on the synthesis problem for a concentric ring array because there is no weight function in Eq. (32). It is worth noting that similar to linear arrays, the reducing procedure of the linear array elements can be easily applied to concentric ring arrays. For this purpose, Eqs. (24), (30) are used to determine the distance between two rings and the number of rings, respectively.

## Results and discussion

In this section, to verify the performance of the proposed method, several practical linear and ring arrays are investigated, and the obtained results are compared.

### Synthesizing of equi-ripple pattern

**Figure 2:**
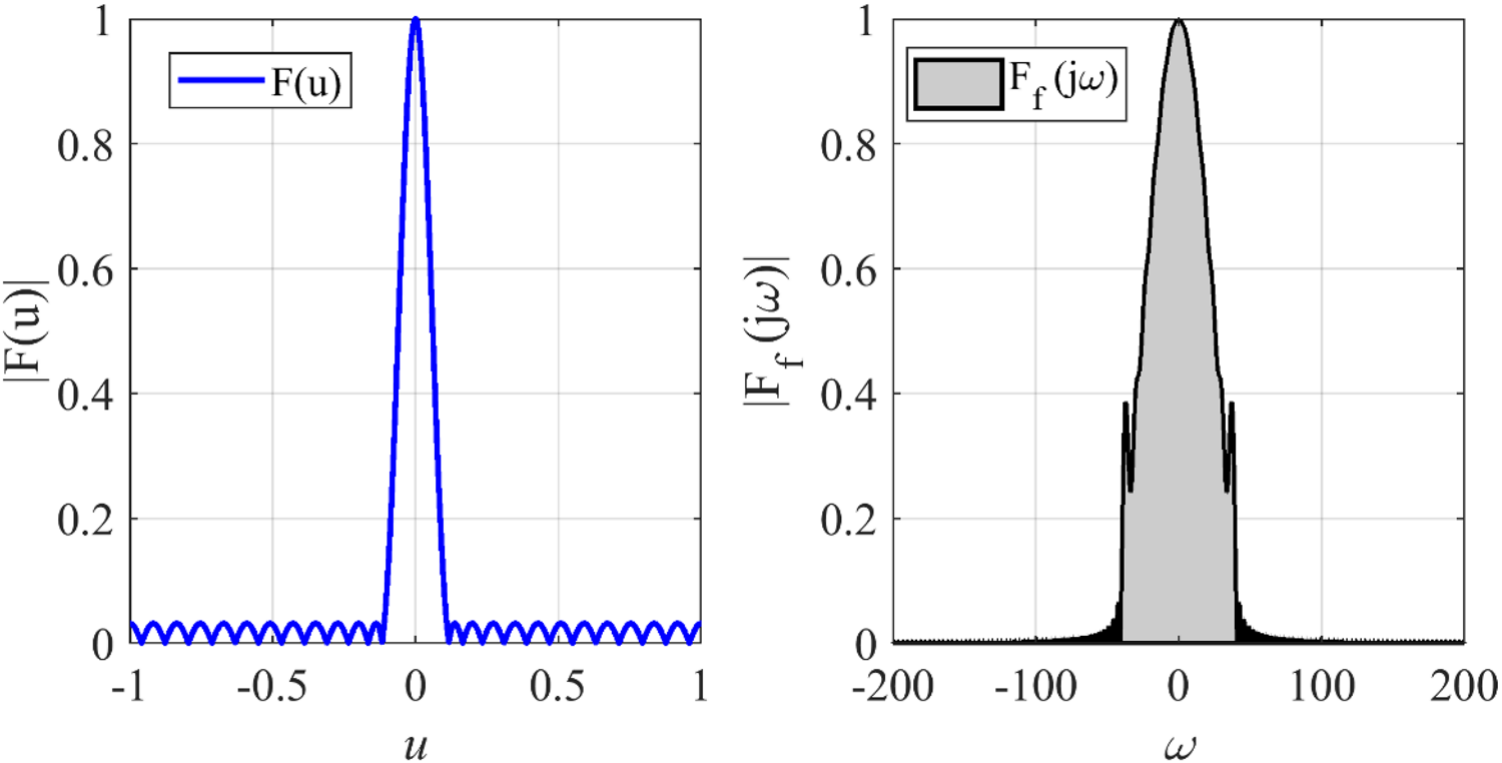
The desired pattern of the first case and its Fourier transform.

**Figure 3:**
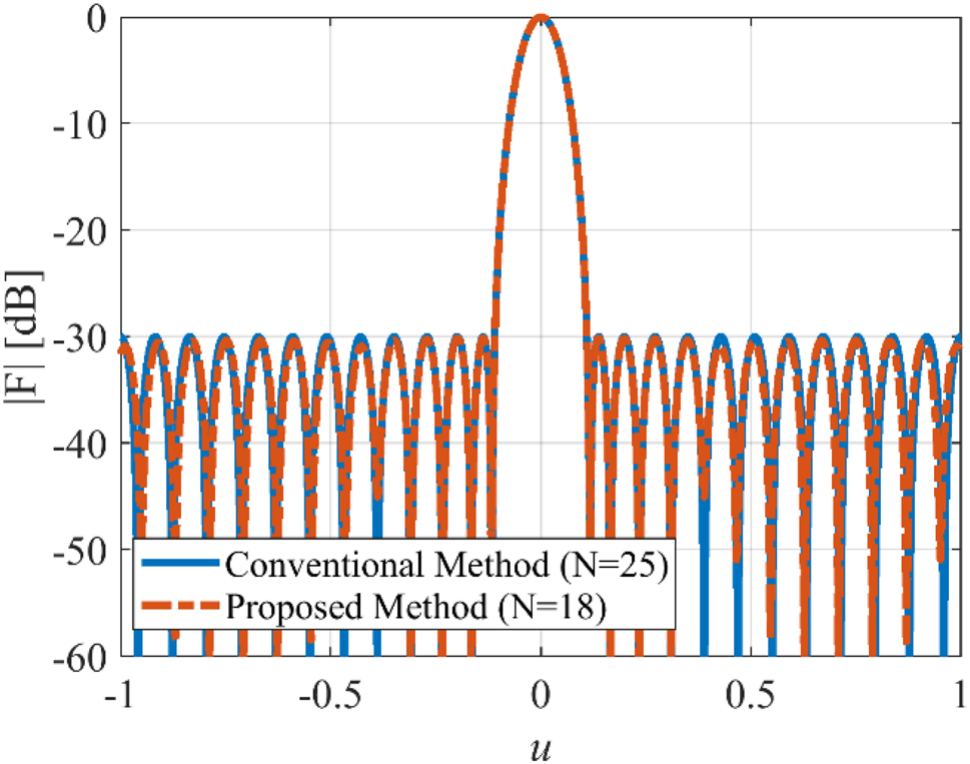
The synthesized results of the first case.

**Figure 4:**
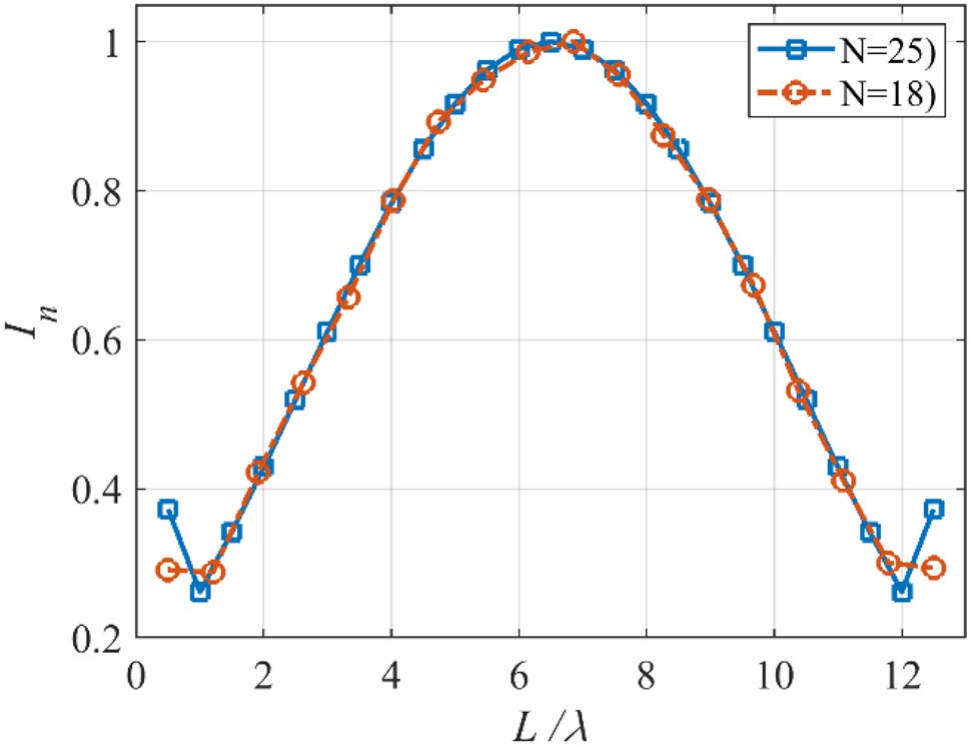
The obtained *I*_*n*_’s of the first case.

The array with equi-ripple pattern is widely used in practical applications. The synthesis method of the equi-ripple pattern was proposed by Dolph and Tschebyscheff^19^. In the first case, we want to show that the prescribed radiation pattern can be synthesized by using a lower number of elements. To this end, we consider an equi-ripple pattern with *SLL* = −30 dB, and array length *L* = 12*λ*. Figure 2 shows the prescribed pattern and its Fourier transform. It is seen that for |*ω*|≤ 57 (*ω*_*M*_≈113), the magnitude of |*F*_*f*_(j*ω*)| is approximately equal to zero. So, according to Eq. (30), the array can be designed with *N* ≥ 18. Assuming *N* = 18, and according to Eqs. (24) and (27), the distance between two adjacent elements is set to *d*≈0.7λ. After applying the proposed method with the mentioned parameters of the array, the obtained results are shown in Fig. 3. It can be seen that the accuracy of the proposed method is approximately at the same level as the result of the Dolph-Tschebyscheff method *N* = 25, *d* = λ/2. In other words, about 28% is saved in the number of array elements by the introduced approach. Also, Fig. 4 shows the obtained excitation coefficients of the proposed and conventional methods.

### Synthesizing of flat-top pattern

**Figure 5:**
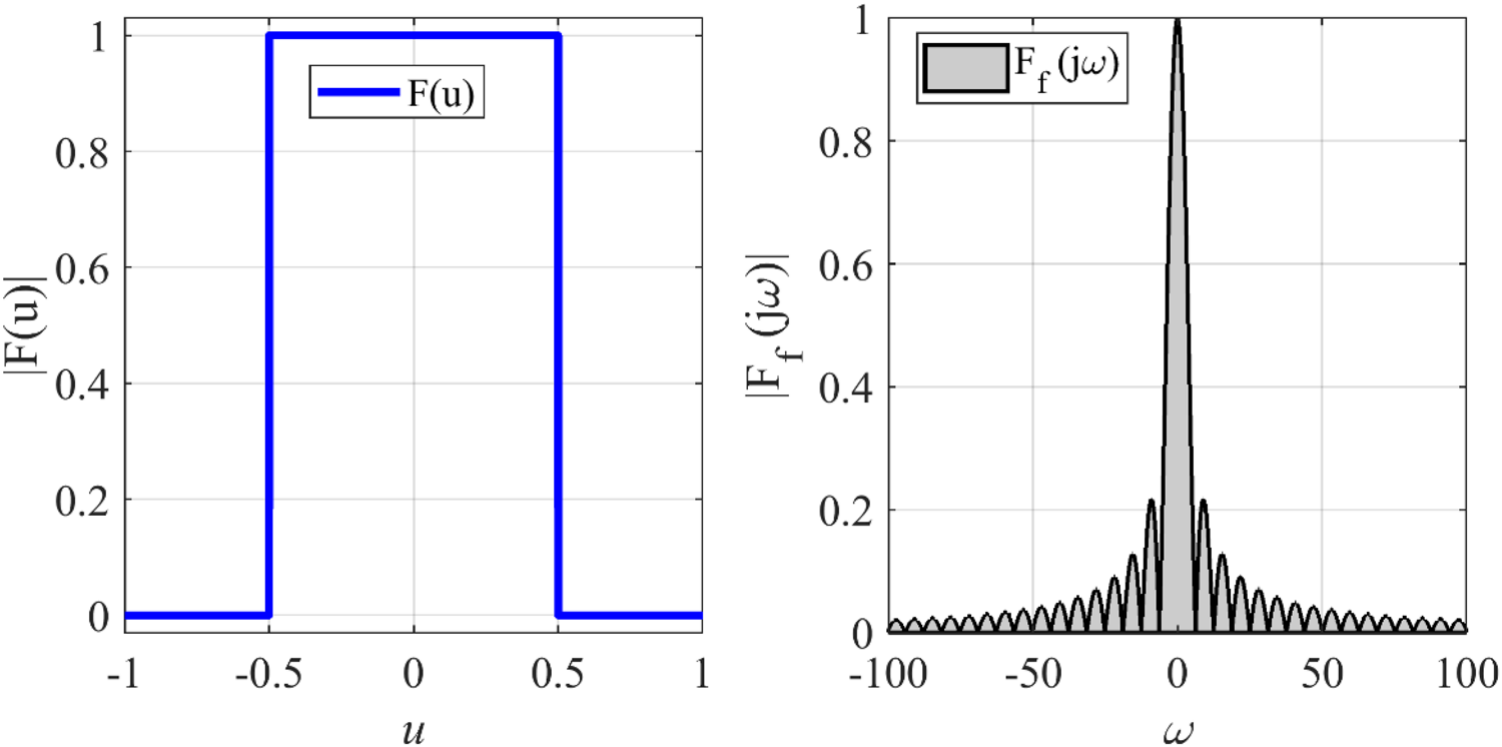
The desired pattern of the second case and its Fourier transform.

**Figure 6:**
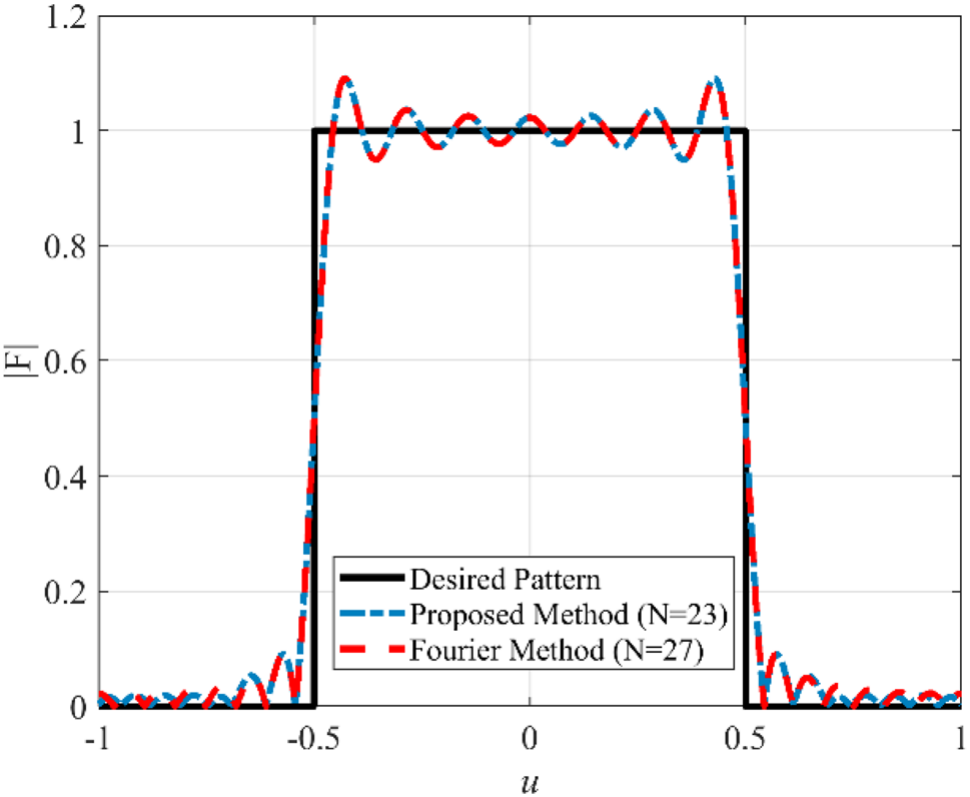
The synthesized results of the second case.

**Figure 7:**
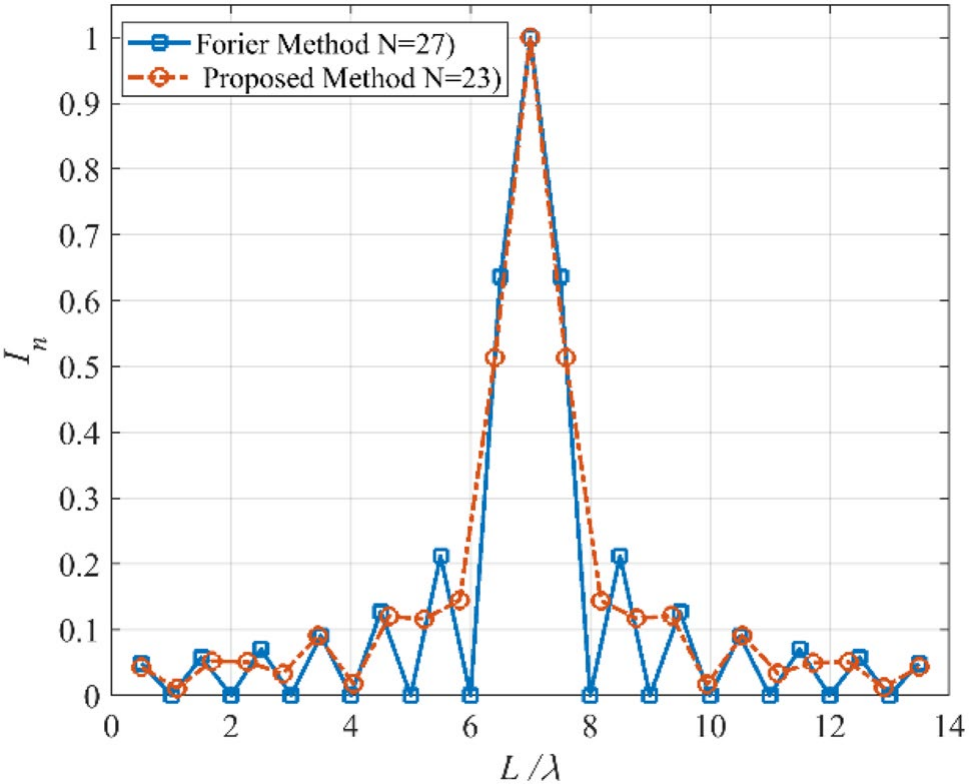
The obtained *I*_*n*_’s of the second case.

Due to several sudden jumps in the radiation pattern, the synthesis of a flat-top pattern is always a challenging problem in antenna engineering. In the second case, the synthesis of a flat-top pattern with non-zero values over the interval |*u*|≤ 0.5 is considered. To this end, the array length is set to *L* = 13*λ*. Figure 5 shows the prescribed pattern and its Fourier transform. The Fourier transform of a flat-top pattern is followed by a Sinc function. It is seen that the magnitude of the Fourier transform of the prescribed pattern is less than 0.02 over the interval |*ω*|≥ 71. So, *ω*_*M*_≈145 is a suitable choice. Although we can choose the higher value for *ω*_*M*_, the obtained result shows that the accuracy will not be changed considerably. Then, the values of *N*, *d* are determined according to Eqs. (30), (24), and (27). The final results can be acquired using the obtained parameters (*N* = 23, *d*≈0.6λ) and applying the Bessel transform technique. Figure 6 shows the obtained result of the proposed method, including the desired pattern. It is seen that the desired pattern can also be synthesized using the other conventional methods like the Fourier method with the same accuracy for *N* = 27, *d* = λ/2. It is seen that the proposed method can achieve an array with a lower number of elements. The obtained excitation coefficients are depicted in Fig. 7.

### Synthesizing of cosecant pattern

**Figure 8:**
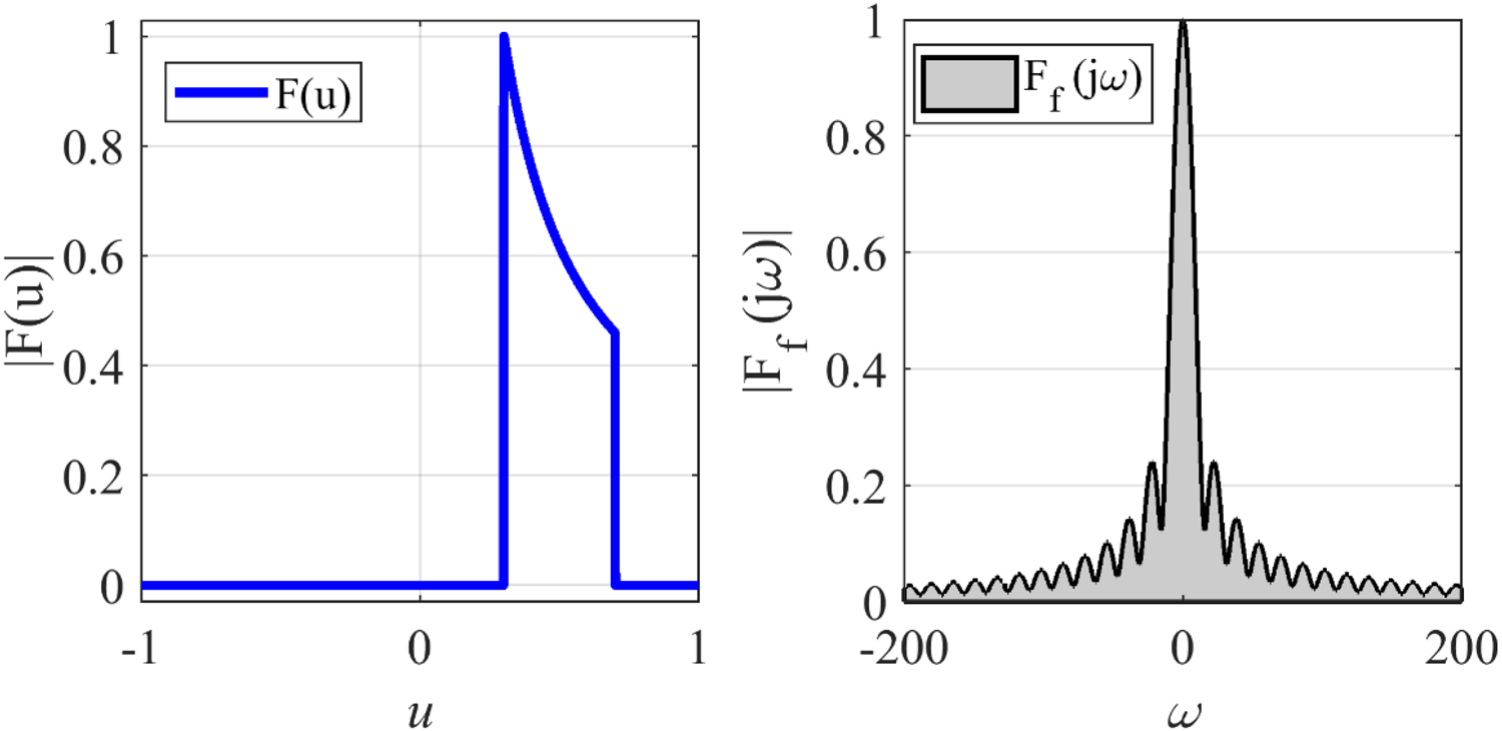
The desired pattern of the third case and its Fourier transform.

**Figure 9:**
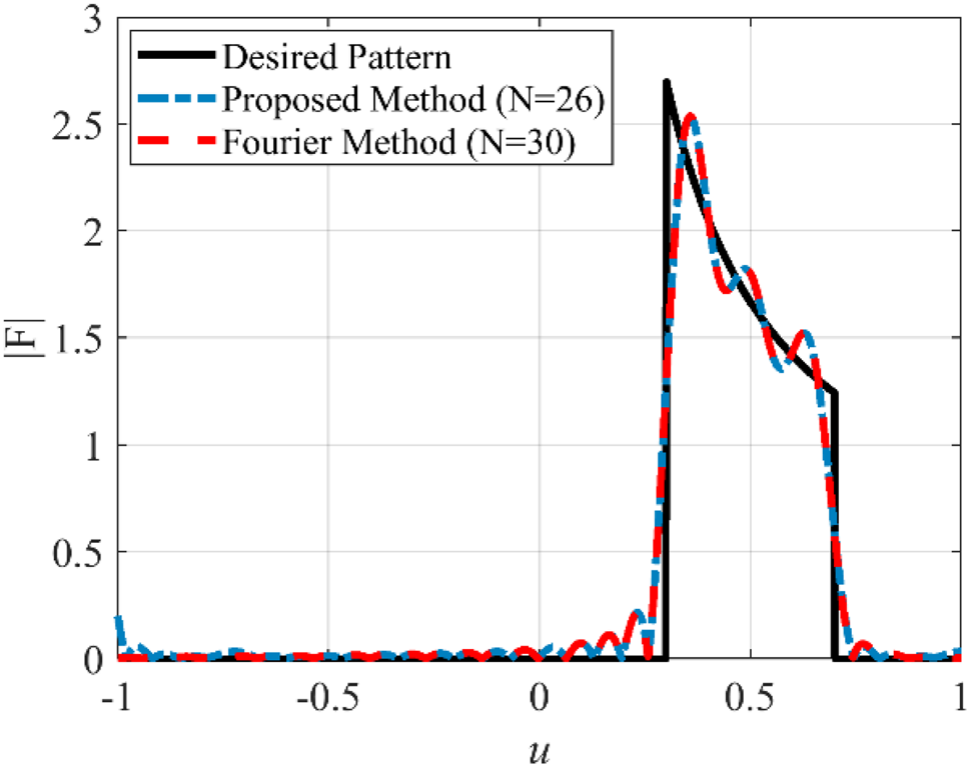
The synthesized results of the third case.

**Figure 10:**
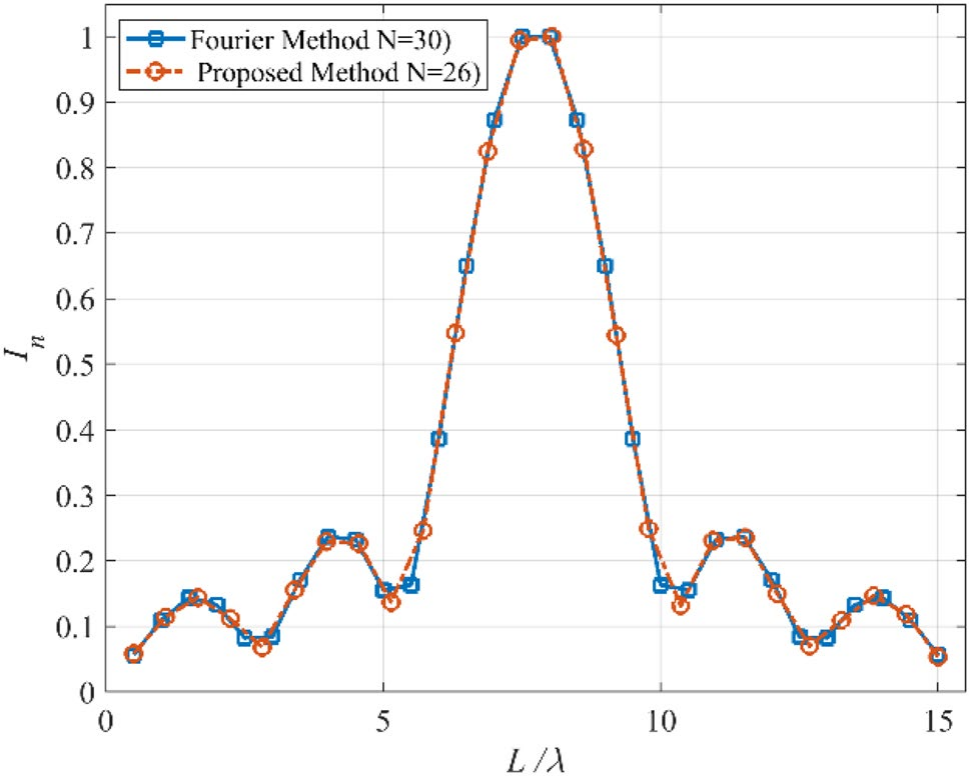
The obtained *I*_*n*_’s of the third case.

An array with a Cosecant radiation pattern is widely used in radar applications. Due to the presence of several sudden jumps in the radiation pattern and non-linear behavior in the main lobe region, the synthesis of a Cosecant pattern, like that of the flat-top pattern, is a challenging problem in antenna engineering. In the third case, synthesis of a Cosecant pattern with non-zero values over the interval 0.3 ≤ *u* ≤ 0.7 is considered. To this end, the array length is set to *L* = 14.5*λ*. Figure 8 shows the prescribed pattern and its Fourier transform. It is seen that for |*ω*|≥ 82, the magnitude of the Fourier transform is ignorable. So, *ω*_*M*_≈162 is an acceptable choice. Using Eqs. (30), (24), and (27), the number of array elements and distance between elements will be *N* = 26, *d*≈0.58λ. Figure 9 shows the obtained result of the proposed method, including the desired pattern. Similar to the previous case, the desired pattern is also synthesized using the Fourier method with parameters *N* = 30, *d* = λ/2. The accuracy of the two methods is comparable, but the introduced approach saves about 28% in the number of array elements. Also, the obtained excitation coefficients are plotted in Fig. 10.

### Synthesizing of wide-null pattern

**Figure 11:**
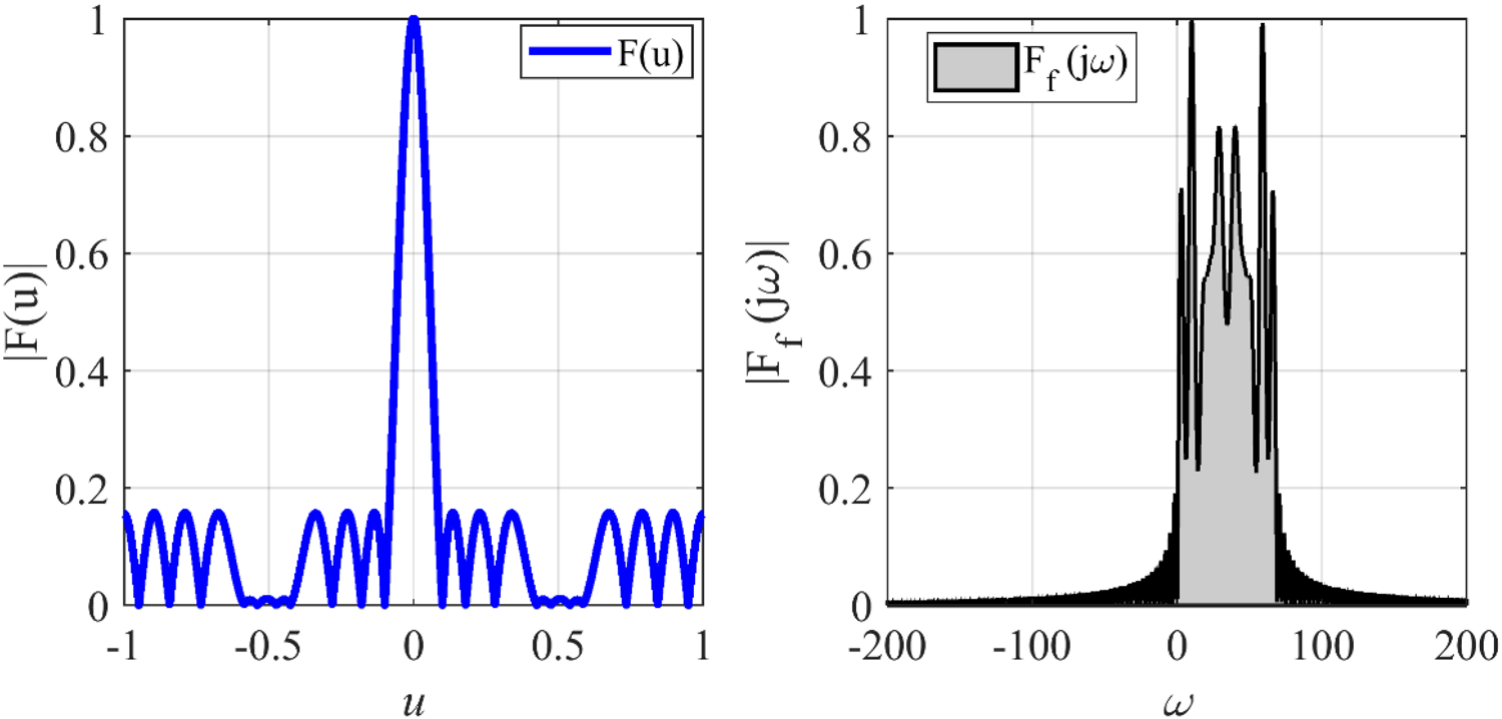
The desired pattern of the fourth case and its Fourier transform.

**Figure 12:**
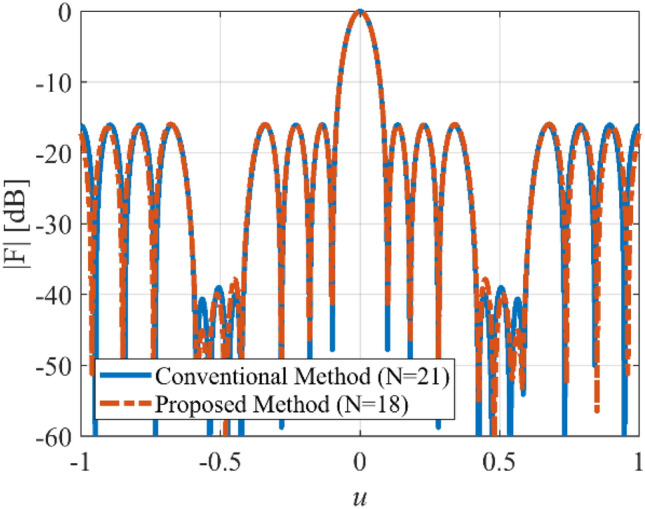
The synthesized results of the fourth case.

**Figure 13:**
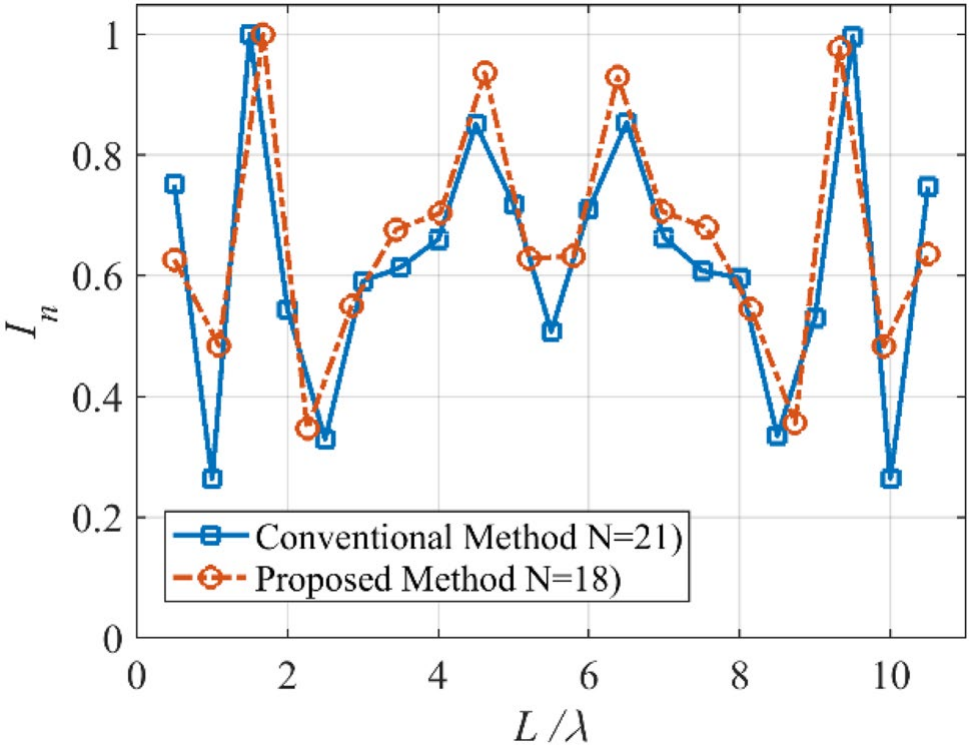
The obtained *I*_*n*_’s of the fourth case.

In tracking applications, to prevent interference, an array with a few wide nulls is required. The numerical synthesis method of the wide-null pattern is proposed in^27^. In the fourth case, we want to determine *N*, *d*, and *I*_*n*_ of a linear array with length *L* = 10λ, and the with wide-null pattern. To this end, we consider an equi-ripple pattern with *SLL* = −15 dB, and two wide nulls about -40 dB. The design procedure of the wide-null array using the numerical technique is discussed in^27^. Figure 11 shows the prescribed pattern and its Fourier transform. It is seen that for |*ω*|≤ 56 (*ω*_*M*_≈113), the magnitude of |*F*_*f*_(j*ω*)| is very low. So, according to Eq. (30), the array can be designed with *N*≈18. By specifying the number of array elements, and according to Eqs. (24) and (27), the distance between two adjacent elements is set to *d*≈0.6λ. After applying the proposed method with the mentioned parameters, the obtained results are shown in Fig. 12. It can be seen that the accuracy of the proposed method is approximately at the same level as the result of^27^ with *N* = 21, *d* = λ/2. In other words, about 14% is saved in the number of array elements by the introduced approach. Also, Fig. 13 shows the obtained excitation coefficients of the proposed and numerical methods.

### Synthesis of concentric ring array

**Figure 14:**
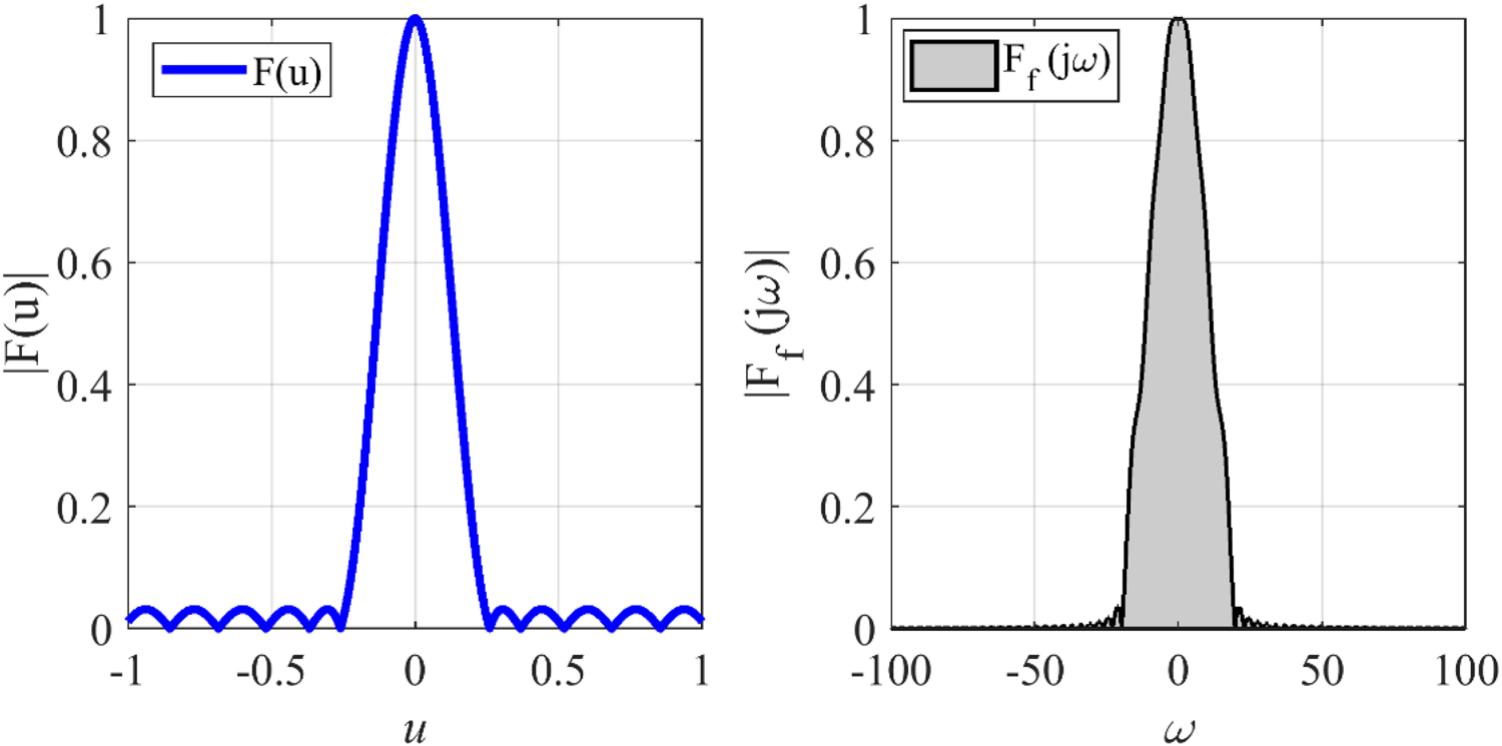
The desired pattern of the fifth case and its Fourier transform.

**Figure 15:**
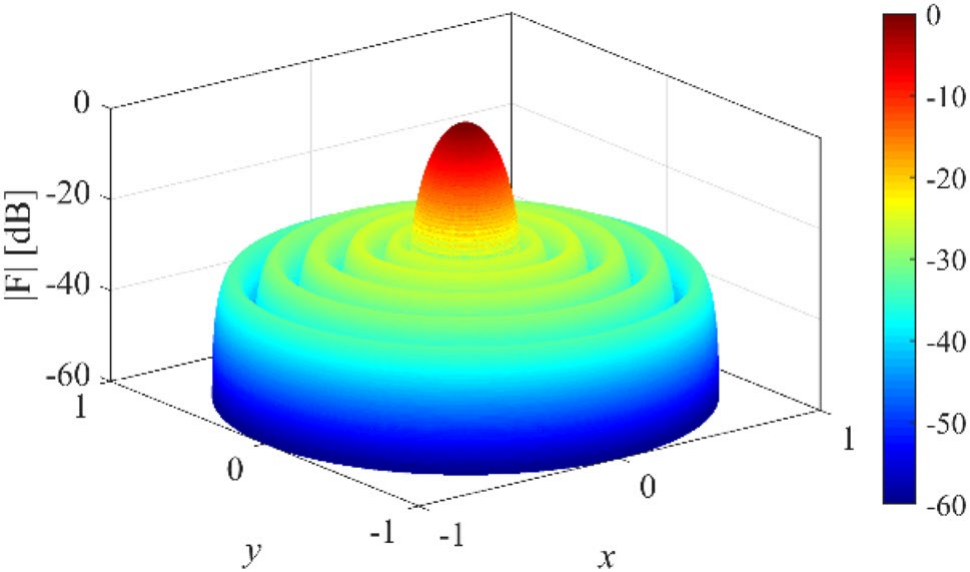
The obtained 3D pattern of the fourth case.

**Figure 16:**
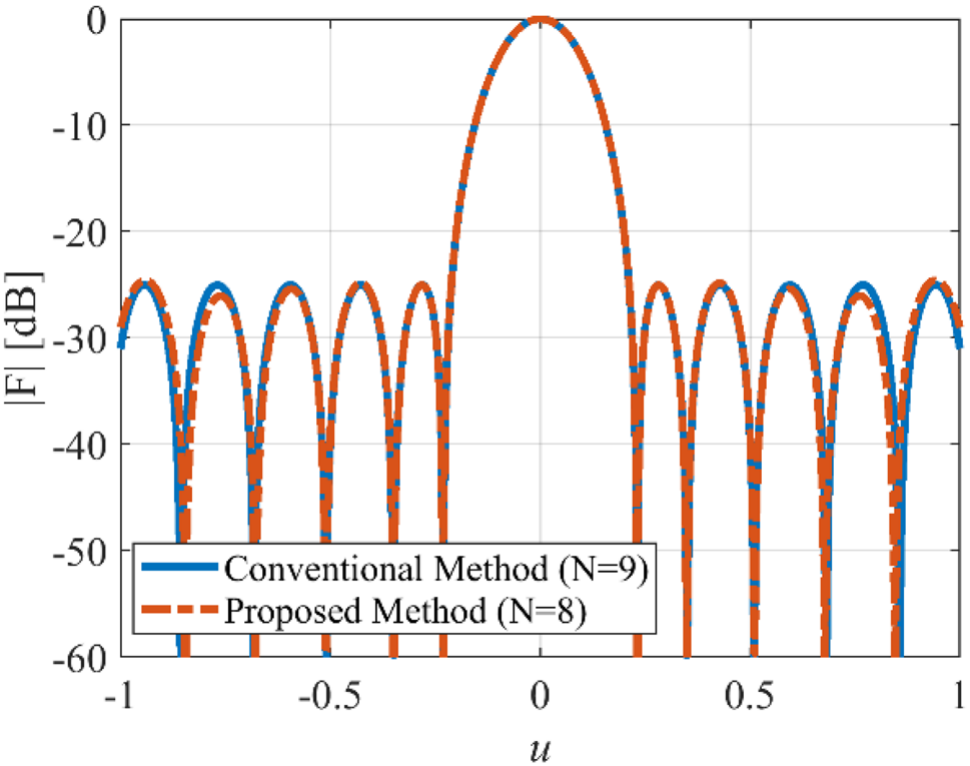
The synthesized results of the fifth case.

**Figure 17:**
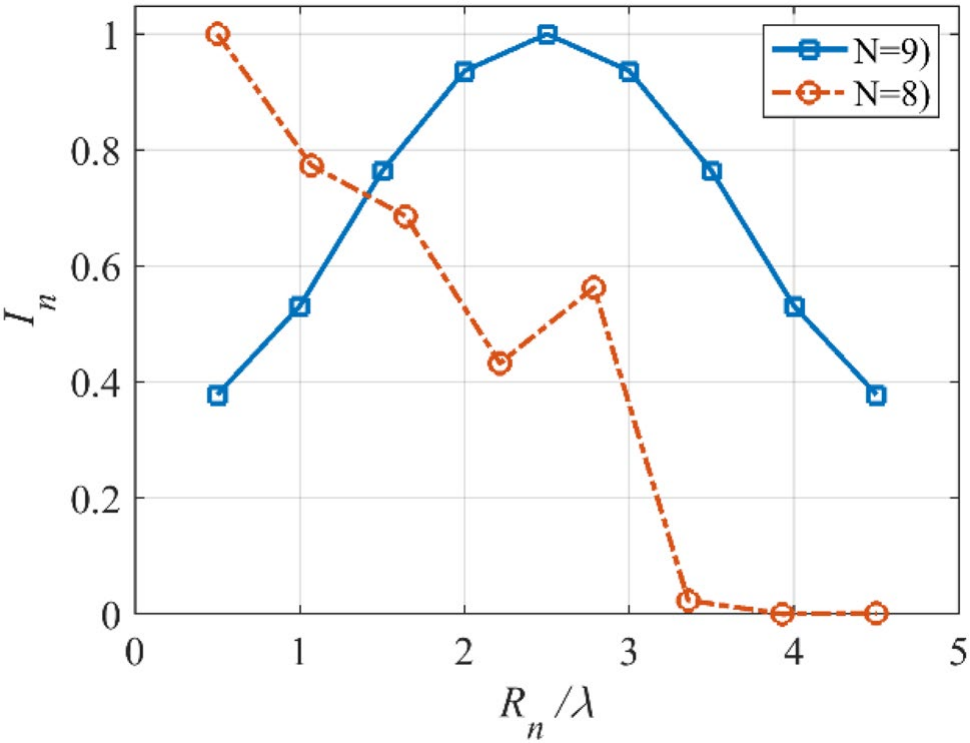
The obtained *I*_*n*_’s of the fifth case.

In the fifth case, we want to design a concentric ring array to have an equi-ripple radiation pattern of about −30 dB. Figure 14 shows the prescribed pattern and its Fourier transform. It is seen that for |*ω*|≤ 25 (*ω*_*M*_≈50), the magnitude of |*F*_*f*_(j*ω*)| is lower than 0.001. So, according to Eq. (30), the concentric ring array can be designed with eight rings (*N*≈8). Then, according to Eqs. (24) and (27), the distance between two adjacent rings is set to *d*≈0.57λ. Three-dimensional radiation pattern of the final array is depicted in Fig. 15. For better comparison, the two-dimensional of the obtained array factor is plotted in Fig. 16. Also, the desired pattern is synthesized using the conventional method with *N* = 9, half a wavelength distance between two rings, and the obtained result is displayed in Fig. 16. It is seen that the accuracy of the two methods is at the same level, and about 20% is saved in the number of array elements by the introduced technique. It should be noted that in a concentric ring array, the total number of array elements is equal to the sum of the elements on all rings. For the under-studying array, the obtained excitation coefficients are plotted in Fig. 17.

For better comparison, some of the important factors are reported in Table 1, including total number of array elements *N*, distance between elements/rings *d*, reduction percent *η*, and mean square error (*MSE*).

**Table 1:** Comparison of the results.

		*N*	*d*/λ	*η* (%)	*MSE*
Case I	Proposed Method	18	0.7059	28	1e-5
	Dolph-Tschebyscheff Method	25	0.5		
Case II	Proposed Method	23	0.5909	15	7.2e-3
	Fourier Method	27	0.5		
Case III	Proposed Method	26	0.5800	13	2.9e-2
	Fourier Method	30	0.5		
Case IV	Proposed Method	18	0.5882	14	2.5e-4
	Method in^27^	21	0.5		
Case V	Proposed Method	226	0.5714	20	1.5e-5
	Method in^26^	283	0.5		

The proposed method is used to design an array with the minimum number of elements. Since reducing the number of elements at a constant length increases the inter-element spacing, the designed array will work well in terms of the mutual coupling effect. Of course, the inter-element spacing is such that the grating lobes will not appear in the final radiation pattern. Also, the computed MSE shows that the accuracy of the final results is in an acceptable range. As a result, the proposed method could be a good candidate for designing linear and concentric ring arrays with optimal parameters.

## Conclusion

The relevant values for the number of array elements and the distance between them should be chosen for equally-spaced arrays. An analytical method based on the Nyquist–Shannon sampling theorem is described in this study for designing an array with the minimum number of array elements and the optimum spacing between elements. It is demonstrated that the required excitation coefficients can be calculated using the Bessel transform of the array factor. The recursive least square method is employed to increase the accuracy of the final solution. As shown, the proposed method can also be easily applied to a concentric ring array. The performance of the introduced method on several practical examples is demonstrated.
